# Learning in Deep Radial Basis Function Networks

**DOI:** 10.3390/e26050368

**Published:** 2024-04-26

**Authors:** Fabian Wurzberger, Friedhelm Schwenker

**Affiliations:** Institute of Neural Information Processing, Ulm University, James-Franck-Ring, 89081 Ulm, Germany

**Keywords:** radial basis function networks, function approximation, function interpolation, classification, Mahalanobis distance, partially connected neural networks

## Abstract

Learning in neural networks with locally-tuned neuron models such as radial Basis Function (RBF) networks is often seen as instable, in particular when multi-layered architectures are used. Furthermore, universal approximation theorems for single-layered RBF networks are very well established; therefore, deeper architectures are theoretically not required. Consequently, RBFs are mostly used in a single-layered manner. However, deep neural networks have proven their effectiveness on many different tasks. In this paper, we show that deeper RBF architectures with multiple radial basis function layers can be designed together with efficient learning schemes. We introduce an initialization scheme for deep RBF networks based on k-means clustering and covariance estimation. We further show how to make use of convolutions to speed up the calculation of the Mahalanobis distance in a partially connected way, which is similar to the convolutional neural networks (CNNs). Finally, we evaluate our approach on image classification as well as speech emotion recognition tasks. Our results show that deep RBF networks perform very well, with comparable results to other deep neural network types, such as CNNs.

## 1. Introduction

The reconstruction of an unknown function based on a finite set of data—typical given as pairs of sensory input and target output—is a major goal in applications of numerical analysis, such as function approximation or pattern classification. In real-world applications, the unknown functions must be modeled using multivariate approximation schemes. Several approaches can be applied for multivariate approximation, such as finite elements techniques or spline functions together with triangulation, just to name a few. Typically, the known dataset is scattered, where it is assumed that the dataset does not have any special properties in terms of density or spacing or other regularities. In [1] Franke discussed the problem of function interpolation given a set of scattered data, and he introduced the basis function approach for such tasks of function interpolation and approximation.

**Figure 1:**
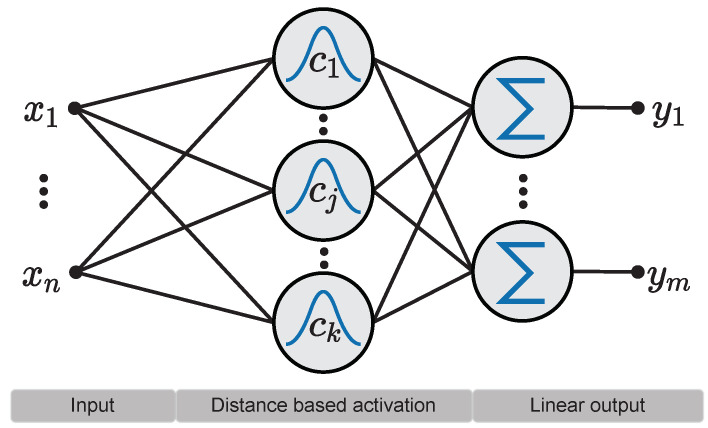
RBF network consisting of a layer of *k* distance based RBF kernels as well as a linear weighted output of the kernels activations. The network consists of *n* input neurons and *m* output neurons.

Radial Basis Functions (RBFs) are a special class of basis functions used for multivariate interpolation of scattered data, where the final interpolating function is obtained by a linear combination of multiple RBF kernel outputs. Kernel functions are of a fixed type, e.g., the Gaussian density function to mention the most common one. In this approach, each RBF kernel calculates its output based on the distance between an input vector and the kernel center by leveraging some pre-defined proximity measure. In terms of artificial neural networks—this approximation scheme is a network with a single layer of RBF kernels, followed by a linear weighting layer. Primary, the Euclidean norm is used for this distance calculation, see Figure 1. More general distance measures such as the Mahalanobis distance have been introduced as proximity measures. This allows for more complex contributions of the input features to the kernel activation. Fundamental mathematical results on RBF can be found in the books by Powell [2], Buhmann [3] or Fasshauer [4], and in the papers authored by Micchelli [5], Dyn [6] or Schaback [7].

Neural network interpretation of RBF networks goes back to Broomhead and Lowe [8] as well as to Moody and Darken [9]. Park and Sandberg [10] proved that RBF networks with one hidden layer are universal approximators, theoretically rendering deeper architectures irrelevant. Broomhead and Lowe uniformly sampled RBF centers from the training data or input space if prior domain knowledge was absent [8]. They optimized the linear output layer directly via the pseudo-inverse solution [8]. Due to the two-layered architecture, the existence of a solution for the optimization problem was guaranteed [8]. Moody and Darken [9] further refined the initialization process by using vector-quantization approaches such as k-means clustering to determine data-based initial center locations [9]. Additionally, they introduced a p-nearest Neighbour (PNN) heuristic to calculate the width parameters used in the Gaussian RBF [9]. The PNN heuristic was chosen to create an overlap between neighboring RBF kernels in order to fully cover the input space [9]. Besides unsupervised clustering, supervised learning vector quantization (LVQ) [11] can be applied to initialize the centers of the RBF centers [12]. Also, ensemble methods [13,14] and semi-supervised learning techniques [15] can be be considered to pre-train an initial RBF architecture. In order to improve the performance of the RBF network, a third optimization phase can be introduced [16]. In this third phase, all network parameters are simultaneously trained by supervised backpropagation training, see Schwenker et al. [16] for details.

Besides this general RBF training procedure a lot of different heuristics have been developed over the years. See, for instance, [17] or [18]. Research was mostly focused on parameter initialization and finding better radial basis functions [19]. However, the same overall network architecture was maintained. More recently, in [19,20] RBF networks were used as classifiers in deep convolutional neural networks while achieving comparable performance to commonly used MLP classifiers on the considered benchmark tasks [19]. The success of deep learning shows that neural networks can benefit greatly from deeper network architectures.

But still, besides the standard deep architectures (using inner product in connection with an increasing nonlinear transformation) there are not many studies on deep networks using distance-based kernels. Results relevant for our work can be found in [21,22,23]. Wenzel et al. introduced the structured deep kernel networks approach in [21], which is similar to the RBF network approach as discussed in our work. They proved analytical approximation properties of the architecture, for instance, the so-called concatenated representer theorem that is a modification of the deep kernel representer theorem given in Bohn et al. [23]. In [21], it is also claimed that their proposed architecture (with unbounded depth) can be asymptotically better than standard neural networks using ReLU activation functions. Furthermore, in [22], Wenzel et al. applied their method successfully to the turbulence closure problem a three-dimensional regression problem.

In our work, we focus more on the problem of high-dimensional input domains for classification problems, (for instance, an input dimension of 784 in case of the MNIST data). In this context, we applied the Cholesky decomposition for positive definite matrices to implement a general learning scheme for the Mahalanobis distance in the RBF kernel approach. All in all, we introduce a deep learning method to (fully and partially connected) radial basis function networks by incorporating data-driven initialization techniques including distance computation for high-dimensional input domains.

The main contributions of this paper can be summarized in the following statements:

1. This paper is not in the mainstream of deep neural network architectures. It is a paper in which we want to describe a new perspective on RBF networks in the context of modern deep learning. This is achieved by proposing an approach that allows the construction and the training of deep networks consisting of RBF units—units that are based on distance computation and kernel functions. In contrast to related work on standard RBF networks, the idea of designing multilayered RBF architectures is considered. Thus, this paper must be considered as an attempt to introduce RBF networks into the field of modern deep learning, with the aim of initiating a discussion on this type of deep and perhaps recurrent type of RBF networks.
2. Moreover, we present in our work a partially connected architecture based on such distance-based RBF units, following the same idea of standard Convolutional Neural Network (CNN) architectures. Thus, this type of architecture may be of interest in applications where typically CNNs are used, for instance, to extract patterns from images.
3. From the implementation point of view we describe how such a distance computation for partially connected units can be implemented in modern deep learning frameworks.
4. In a series of experiments, we investigated the use of RBF-based deep architectures on benchmark applications.
5. This paper is a proof of concept to train deep RBF networks for high-dimensional input domains. Of course, such deep RBF networks need more research. In particular, research on different types of activation functions, distance functions, as well as parameter initialization and parameter optimization is necessary—it is a very first step and may lead to a new direction of research in deep RBF architectures.

The remainder of this work is structured as follows. In Section 2, we give a short introduction to shallow RBF networks and describe the main components and methods necessary to train deep RBF networks. Then, we briefly describe our experimental framework as well as report the results of our experiments in Section 3. Finally, we discuss our results in Section 4, followed by a conclusion and a proposal for possible future research in Section 5.

## 2. Deep RBF Networks

In this work, we focus on multi-layer RBF networks, which we call deep RBF networks. We keep the overall RBF architecture consisting of an input layer and a linear output layer. In contrast to regular RBF networks we allow for multiple hidden layers with RBF kernels. Finally, we distinguish between fully connected and partially connected RBF layers. In the latter we follow a similar approach to CNNs, resulting in a patch-wise distance calculation for the RBF kernels. We use partially connected layers to extract features, followed by a regular (shallow) fully connected RBF network for classification.

### 2.1. Shallow RBF Networks

A typical shallow RBF network consists of a single layer of RBF kernels, followed by a fully connected linear layer. The linear layer combines the output of the RBF kernels based on the weight matrix W:

(1) $$y_{j}\left(x\right)=\sum_{i=1}^{k}w_{ij}\cdot h_{i}\left(x\right)$$

The overall architecture is shown in Figure 1. The kernel activation $h_{i}\left(x\right)$ of neuron *i* is calculated by the squared Mahalanobis distance between center $c_{i}$ and the input vector x, followed by an activation function *f*:

(2) $$h_{i}\left(x\right)=f\left({∥x-c_{i}∥}_{R_{i}}^{2}\right)$$

The Mahalanobis distance is specified by a positive-definite matrix $R_{i}$ with

(3) $${∥x-c_{i}∥}_{R_{i}}=\sqrt{{\left(x-c_{i}\right)}^{T}R_{i}\left(x-c_{i}\right)}$$

We call $R_{i}$ the Mahalanobis distance matrix in the following. In contrast to other neural networks, RBF networks use a distance based activation provided by the RBF kernels. Commonly, the Gaussian function is used as activation function. A selection of activation functions used for RBF networks is given below, where *r* is the squared Mahalanobis distance:

(4) $$\begin{array}{cc} \mathrm{Gaussian} & \;\;h\left(r\right)=e^{-r} \end{array}$$

(5) $$\begin{array}{cc} \mathrm{Quadratic} & \;\;h(r)=1-r \end{array}$$

(6) $$\begin{array}{cc} \mathrm{Multiquadric} & \;\;h\left(r\right)=\sqrt{1+r} \end{array}$$

(7) $$\begin{array}{cc} \mathrm{Inverse}\;\mathrm{quadratic} & \;\;h\left(r\right)=\frac{1}{1+r} \end{array}$$

(8) $$\begin{array}{cc} \mathrm{Inverse}\;\mathrm{multiquadric} & \;\;h\left(r\right)=\frac{1}{\sqrt{1+r}} \end{array}$$

### 2.2. Partially Connected RBF Networks

**Figure 2:**
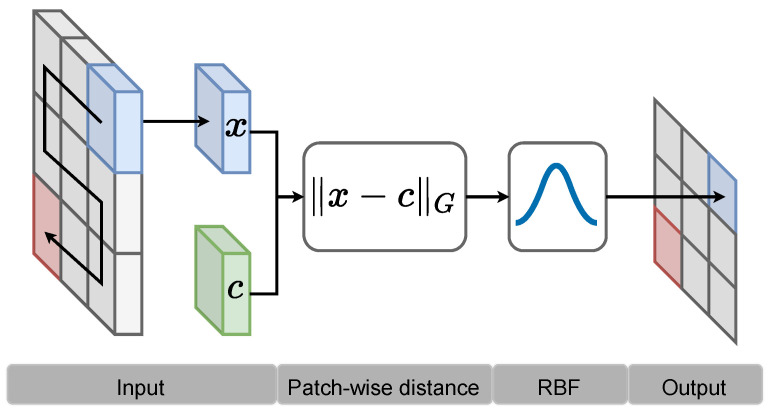
Patch-wise distance calculation, followed by a RBF.

We consider the context of image classification with 2D inputs. Given an input image $X\in R^{W\times H\times C}$ where *W* and *H* denote the image width and height and *C* the channel dimension. For partially connected layers we follow the idea of the weight sharing approach as used in CNNs. The activation map $h\left(X\right)\in R^{W\times H\times 1}$ of a single RBF neuron can be calculated as follows:

(9) $$h\left(X\right)=f\left(\frac{1}{C}\sum_{c=1}^{C}{∥X_{c}⊖c_{c}∥}_{R_{c}}^{2}\right)$$

with center $c\in R^{N\times N\times C}$ and Mahalanobis distance matrix $R_{c}\in R^{N^{2}\times N^{2}\times C}$. The ⊖ operation denotes patch-wise subtraction in a sliding window similar to a convolution operation. Figure 2 illustrates the general idea. A more detailed mathematical description is given below.

Note that in the above formula padding and strides are omitted. As higher numbers of input channels increase the sum, we normalize the sum by $\frac{1}{C}$ before applying the RBF. Otherwise the output activations tend to zero with increasing number of channels.

### 2.3. Cholesky Decomposition

We use the Mahalanobis distance as distance metric between centers and data points in the RBF kernel. This metric, represented by a Mahalanobis distance matrix, is adapted to the dataset at hand. To ensure at least positive semi-definiteness during training we use the Cholesky decomposition on the Mahalanobis distance matrix $R_{j}$ via a lower triangular matrix $G_{j}$ in the following way:

(10) $$\begin{array}{c} R_{j}=G_{j}G_{j}^{T} \end{array}$$

and then optimize only the triangular matrix $G_{j}$. This results in an alternative formulation of the squared Mahalanobis distance between input x and center $c_{j}$:

(11) $$\begin{array}{cc} {∥x-c_{j}∥}_{G_{j}}^{2} & ={\left(x-c_{j}\right)}^{T}G_{j}G_{j}^{T}\left(x-c_{j}\right)\\ \; & ={\left[G_{j}^{T}\left(x-c_{j}\right)\right]}^{T}G_{j}^{T}\left(x-c_{j}\right)\\ \; & =∥G_{j}^{T}\left(x-c_{j}\right){∥}_{2}^{2} \end{array}$$

### 2.4. Patch-Wise Distance Calculation as Convolution

Calculating the Mahalanobis distance between image patches and centers is an expensive operation. We reformulate the problem in terms of convolutions, which are efficiently implemented in most machine learning frameworks. In the following equations, the indices indicating individual neurons and channels are omitted. Additionally, the convolution operator ∗ is used.

#### 2.4.1. Euclidean Distance

The Euclidean distance between an image patch x and a center c can be reformulated in the following way:

(12) $$\begin{array}{cc} {∥x-c∥}^{2} & =\sum_{i=1}^{N}{\left(x_{i}-c_{i}\right)}^{2}=\sum_{i=1}^{N}x_{i}^{2}-2x_{i}c_{i}+c_{i}^{2}\\ \; & =\sum_{i=1}^{N}x_{i}^{2}+\sum_{i=1}^{N}c_{i}^{2}-2\langle x,c\rangle \end{array}$$

The last dot product translates into a convolution when performing the calculation in a sliding window fashion. This results in an efficient implementation of the scaled Euclidean distance. In this case, we can assume that G is a diagonal matrix with diagonal elements σ reshaped to a matrix $\sigma \in R^{N\times N}$:

(13) $$\begin{array}{c} {∥X⊖c∥}_{G}^{2}=\sigma ⊙{∥X⊖c∥}^{2}=\left(X^{⊙2}*\sigma \right)-2\left(X*\left(\sigma ⊙c\right)\right)+\sum_{i=1}^{N}\sum_{j=1}^{N}c_{ij}^{2}\sigma_{ij} \end{array}$$

where ^⊙2^ denotes element-wise squaring, ⊙ is the Hadamard product and ⊖ is the patch-wise subtraction, performed over a sliding window.

#### 2.4.2. Mahalanobis Distance

The calculation of the Euclidean distance is simple due to the decomposition into a dot product and square terms. This is a direct result of the Euclidean distance being characterized by a diagonal distance matrix. For the Mahalanobis distance with the arbitrary distance matrix, the covariance between feature dimensions has to be considered. We derived the following simplification, consisting of multiple stages of convolution operations. Let $X\in R^{W\times H}$ denote a single-channel input image, $G\in R^{N^{2}\times N^{2}}$ the Cholesky decomposed distance matrix and $c\in R^{N\times N}$ the corresponding neuron center. An image patch $p_{ij}$ at location (i,j) is defined as follows:

(14) $$\begin{array}{c} p_{ij}=\left[\begin{array}{ccc} x_{ij} & \cdots & x_{i,j+N-1}\\ \vdots & \ddots & \vdots \\ x_{i+N-1,j} & \cdots & x_{i+N-1,j+N-1} \end{array}\right]\in R^{N\times N} \end{array}$$

In the following, the centers and image patches are used in their flattened representation, where all rows of the corresponding matrix are concatenated. This is denoted as $\bar{c}=\left[\begin{array}{c} c_{11},\ldots ,c_{1N},c_{21},\ldots ,c_{NN} \end{array}\right]$. For the Mahalnobis distance to a single image patch it holds that

(15) $$\begin{array}{cc} G^{T}\left(\bar{p_{ij}}-\bar{c}\right) & =\left[\begin{array}{c} g_{11}\left(x_{ij}-c_{11}\right)+\cdots +g_{1,N^{2}}\left(x_{i+N-1,j+N-1}-c_{NN}\right)\\ \vdots \\ g_{N^{2},1}\left(x_{ij}-c_{11}\right)+\cdots +g_{N^{2},N^{2}}\left(x_{i+N-1,j+N-1}-c_{NN}\right) \end{array}\right]\\ \; & =\left[\begin{array}{c} {\bar{p_{ij}}}^{T}g_{1*}-{\bar{c}}^{T}g_{1*}\\ \vdots \\ {\bar{p_{ij}}}^{T}g_{N^{2}*}-{\bar{c}}^{T}g_{N^{2}*} \end{array}\right] \end{array}$$

For the whole image, the dot products are replaced by convolution operations, resulting in the following patch-wise distance calculation:

(16) $$\begin{array}{c} {∥X⊖c∥}_{G}^{2}=\sum_{i=0}^{N}{\left(X*{\hat{g}}_{i*}-{\bar{c}}^{T}g_{i*}\right)}^{⊙2} \end{array}$$

where ${\hat{g}}_{i*}\in R^{N\times N}$ denotes the *i*-th row of G, viewed as a square matrix. Note that each row of the Cholesky decomposed matrix G has to be convolved with the input image. With the increasing kernel size *N* and larger input images, this calculation may be slow and memory intensive.

### 2.5. Parameter Initialization

We follow commonly used strategies to initialize the centers and matrices. The centers are initialized by k-means clustering while the distance matrices are initialized by a PNN heuristic. To initialize subsequent layers we use a cascading scheme where we initialize the parameters of a layer with the output of the previous layer. To initialize the whole network, the input data are propagated through all layers. During initialization, the last classification layer can be viewed as a single linear transformation with a fixed input. Thus, we can make use of the pseudo-inverse solution to initialize the weights of the linear output layer.

#### 2.5.1. k-Means Clustering

The centers $c_{j}$ of a single neuron *j* are initialized by k-means clustering on the corresponding input data. Clustering can be performed in an unsupervised manner, where global statistics of the input are extracted as centers. In this case, non-labeled data can be used to find good initial parameters. Additionally, class labels can be leveraged in the clustering process by performing the clustering process on data of a given class. This results in more class specific statistics as initial centers. We refer to this approach as *class-specific k-means* clustering in contrast to *global k-means* clustering, where the centers are selected by clustering the whole input data. An advantage of class-specific k-means may be that the number of centers per class can be adjusted to, e.g., counteract underrepresented classes. However, a heuristic is needed to determine the number of selected centers per class. For very small datasets, the clustering approach is not possible when using more neurons than distinct clusters, limiting the number of obtainable initial centers. To speed up the clustering process we use mini-batch k-means clustering as proposed in [24].

#### 2.5.2. Patch-Wise k-Means Clustering

For the partially connected networks, we initialize the centers by extracting image patches from all training samples and performing global or class-specific k-means clustering on those patches. Patches are extracted by using a sliding window over the training samples. Each input image generates several image patches, resulting in a large number of image patches.

Additionally, processing of individual input channels has to be considered. One possibility to address this issue is to perform clustering for each channel individually. However, with the increasing number of input channels this approach is not feasible. We propose to view an input image together with its channels as a volume and perform clustering over this volume. This approach is fast but leads to correlated channels, which may reduce model flexibility.

#### 2.5.3. Mahalanobis Distance Matrix Initialization

Initially, the distance matrix is approximated by the following *p* nearest neighbour PNN heuristic on the centers:

(17) $$\begin{array}{c} \sigma_{j}=\frac{\alpha }{p}\cdot \sqrt{\sum_{i\in N}{∥c_{j}-c_{i}∥}^{2}} \end{array}$$

where N contains the *p* nearest neighbors of center $c_{j}$, based on the Euclidean distance. The parameter α is a scaling factor which has to be set heuristically. The goal of this heuristic is to achieve a good coverage of the input space by adjusting the kernel widths $\sigma_{k}$ as proposed by [9]. This PNN heuristic is fast to calculate as it is solely based on the centers and does not depend on the number of training samples. However, the heuristic fails for non-distinct or too similar centers, where the distance approaches zero. In this case, we set $\sigma_{j}=1$, which results in an Euclidean distance matrix. This approach yields a diagonal matrix with width parameters for each input dimension on the diagonal:

(18) $$\begin{array}{c} G_{j}=\frac{1}{\sigma_{j}}\cdot I \end{array}$$

### 2.6. Training Procedure

After initialization, the full network is trained by back-propagation. We use a softmax activation in the final linear layer in combination with the cross-entropy loss for classification.

For the Mahalanobis distance matrices, training can be carried out either on the full Cholesky decomposed matrix, i.e., on the lower triangular matrix $G_{j}$, or to reduce complexity only on the diagonal entries of the triangular distance matrix. The latter implicitly forces the assumption that the feature dimensions are uncorrelated by ignoring the covariance, resulting in a feature-wise scaled version of the Euclidean distance. The number of parameters used for the Cholesky decomposed matrix (which is triangular) and a single neuron with input dimensionality *C* is $\frac{C(C+1)}{2}$. Using the diagonal entries of the Mahalanobis distance matrix reduces the number of parameters to *C*, which improves training speed.

Additionally, we propose a supervised approach to pre-train subsequent layers by temporarily adding a fully connected softmax layer for classification and minimizing cross entropy loss, virtually resulting in training a single layer RBF network for each layer.

To get an impression of how good individual classifiers perform, we further combine the prediction of multiple classifiers as follows: Each classifier makes an individual prediction, which is selected by its certainty. The certainty is based on the output of the softmax layer. Predictions with a certainty above 95% are summed and the final prediction is obtained by an argmax operation on the summed output distribution.

## 3. Experimental Evaluation

Similar to CNNs, our architecture makes use of data which are strictly arranged on a uniform grid like images. Thus, we evaluate our approach only on 2D structured data. Namely, we use common image classification tasks as well as speech emotion recognition tasks where we pre-process the speech data into MEL spectrograms of a fixed size.

**Figure 3:**
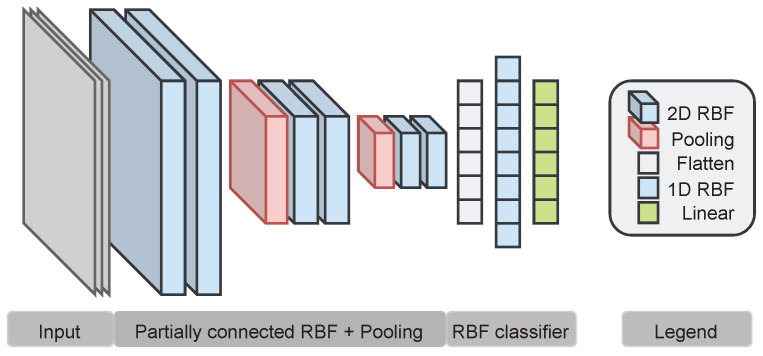
Full RBF architecture used for image classification and emotion recognition.

For both types of tasks, we use the network architecture depicted in Figure 3, consisting of partially as well as fully connected RBF kernels. The Gaussian kernel function is used as an activation function in all experiments. We consider single classifiers and ensemble classification of 5 classifiers trained on different parameter initializations. For the ensembles, the best classifiers are selected according to low entropy in their prediction (i.e., maximum class probability >95%). The prediction of those classifiers is aggregated by summation and finally the class with the highest value is selected. Note that the entropy selection mechanism does not consider the correctness of the prediction.

Task-dependent details and hyperparameters are given in the corresponding section.

### 3.1. Datasets

In the following, we describe the used datasets in more detail. For image classification, we include the common benchmark datasets *MNIST* [25] and *CIFAR10* [26]. For both datasets, we first scale the images to the interval [0,1]. Besides classic image classification benchmarks, we also include the following two speech emotion recognition datasets for evaluation: *RAVDESS* [27] and *EmoDB* [28].

**MNIST** is a gray-scale image dataset often used to evaluate machine learning models. The dataset consists of 28×28 pixel-sized images of hand-written digits from zero to nine, resulting in a 10-class classification problem. In total, there are 60,000 training samples and 10,000 test samples.

**CIFAR10** consists of RGB images from ten different classes, containing different objects and animals. Each image has 32×32 pixels, resulting in 3072 features per image. The whole dataset is similarly sized to the MNIST dataset, containing 50,000 training samples and 10,000 test samples.

**EmoDB** is a small emotion recognition dataset where neutral sentences are spoken by ten different actors in seven different emotions. The following emotions are contained in the data: anger, boredom, anxiety, happiness, sadness, disgust, and neutral. In total, the dataset consists of 535 speech recordings. As the dataset is rather small, we also consider a simpler binary classification task based on arousal. For this task we define anger, anxiety, happiness, sadness, and disgust as emotions with high arousal. Boredom, sadness, and neutral are defined as emotions with low arousal. We reserve 20% of the samples for testing and split the remaining data into 20% validation and 80% training data.

**RAVDESS** is an acted emotion dataset consisting of speech, song, and videos with speech. In total there are 24 actors which speak and sing sentences of the form “Kids are talking by the door” in 8 different emotions. Additionally, portrait videos of the actors speaking the same sentences are available. The following eight emotions are included: neutral, calm, happy, sad, angry, fearful, disgust, and surprised. Note that there are different emotions than in the EmoDB dataset. More precisely, boredom and anxiety is missing in the RAVDESS dataset, while calm and surprised occurs only in the RAVDESS dataset. In total the dataset consists of 2452 sound files, where 1440 are spoken sentences files and the remaining 1012 are sung sentences. The video files are omitted for our tasks as they contain the same audio as the spoken sentences. We use 20% of the data for testing and split the remaining data into 20% validation and 80% training data.

### 3.2. Image Classification

**Architecture** Our image classification architecture is composed as follows. First, we build blocks of two partially connected layers each, followed by a max pooling operation to reduce the spatial dimensions. In total, we use three such blocks with 20 RBF kernels in each block. All kernels use the full Mahalanobis distance matrix and are initialized with class-specific k-means clustering. Finally, a RBF network with 128 neurons is used for classification. The classification network uses the Euclidean distance, which allows for more individual neurons compared to the Mahalanobis distance, while keeping the number of parameters low. This is necessary as the input dimension to the classifier is rather large, resulting in a large number of parameters when using the Mahalanobis distance.

**Training** We use the Adam optimizer in its standard configuration for all of our experiments. We train our models on augmented data, where we shift, scale, flip, and rotate the input images to generate more training data. The whole network is trained for 200 epochs, individual layers are not pre-trained.

**Results** The results for the MNIST dataset are summarized in Table 1. In average, our RBF architecture reaches a test accuracy of 99.5% with a low standard deviation. Using 5 classifiers as an ensemble further increases the test accuracy to 99.67%. Nonetheless, the performance is not on-par with state-of-the art CNN architectures, achieving over 99.8% with a similar number of parameters.

In Table 2, the results for the CIFAR10 dataset are summarized. Our RBF approach reaches a mean test accuracy of 80.72% with a rather high standard deviation of 0.63%. Performing an ensemble classification with again 5 classifiers improves the performance to 85.01%, similar to early CNN architectures like [29]. Note that more recent architectures are able to reach test accuracies beyond 99% on CIFAR10 [31,32].

### 3.3. Speech Emotion Recognition

**Architecture** Our emotion recognition RBF networks consist of 4 partially connected layers with 8 neurons each, followed by a RBF classifier with 128 neurons. The centers are initialized with global k-means clustering. We use global k-means clustering as we only use 8 kernels, while distinguishing 7 different classes. Due to the high input dimensions, more kernels would increase the number of parameters which we aim to keep low.

**Training** We first extract the MEL spectrogram-based features from the audio files for both datasets. We limit the samples to a duration of three seconds and use use 128 filters for the MEL spectrogram. In total, our pre-processing yields a 128×256 feature map for each speech sample. As both emotion datasets are rather small, we augment the spectrograms by randomly shifting the whole spectrogram in the time domain as well as masking random parts of the frequency and time domain as proposed by [34]. The network is optimized by using the Adam optimizer with default parameters. The whole network is trained for 200 epochs without pre-training individual layers.

**Results** In all three instances of the two multi-class emotion recognition tasks, we observe a high standard deviation concerning the test accuracy. For the EmoDB dataset (see Table 3), we achieve a mean test accuracy of 72.15% and 74.76% for a single classifier and ensemble classification, respectively. Constraining the task to a binary problem gives more consistent results with a mean test accuracy of over 96% and a lower standard deviation. Those results seem consistent with a CNN approach on the same MEL spectrograms [35] reaching 72.06% test accuracy. More sophisticated approaches like [36] are able to achieve even better results. Regarding the RAVDESS dataset (see Table 4), we observe similar performance as the considered CNN architectures [37,38] while using significantly fewer trainable parameters. Namely, we reach a test accuracy of 71.41% and 74.30% for a single classifier and ensemble classification, respectively.

Including the song samples for the RAVDESS dataset further improves the testing accuracy on the combined dataset to 78.82%.

## 4. Discussion

A common observation between all evaluated datasets is the increased performance when considering ensemble classification of multiple RBF classifiers. This indicates that the full potential of our approach is not yet reached when considering single classifiers. This assumption is also reinforced by the high variance in test accuracy of single classifiers.

For the emotion recognition datasets, the variance in test accuracy is higher than for the image classification tasks. We attribute this behavior to the low number of training samples in those datasets.

Note that for the emotion recognition tasks the comparison between different models is complicated due to inconsistent feature selection and evaluation procedures. CNN14 and ERANN use four-fold cross-validated results [37,38] while we only report the mean test accuracy over a fixed test set (containing 20% of the samples). Overall we observe a similar performance between our approach and the well-established CNN architectures on all considered datasets, which are promising.

## 5. Conclusions

In this paper, a new way of using Gaussian RBF kernels in deep neural networks is discussed by introducing an initialization scheme suitable for multi-layered RBF architectures. In addition, a partially-connected RBF layer similar to CNN architectures was studied. By utilizing the Cholesky decomposition, we guarantee positive semi-definiteness of the learnable distance matrix used in the Mahalanobis distance calculation. We showed that our proposed architecture performs well on 2D-structured data like images and MEL spectrograms. In contrast to the CNN, the distance-based activation used in our RBF kernels favors interpretability of the underlying calculation. Nonetheless, the flexibility of the proposed deep RBF approach—for instance, due to the Mahalanobis distances offering complex approximations of data densities—can be a disadvantage in other contexts, such as limited scalability to larger network architectures.

All in all, the proposed deep RBF architecture is just the very first step in this direction of research and several aspects of learning in deep RBF networks must be left to future research, e.g., we focus on the Gaussian as a representative of the RBF kernels for several reasons: Gaussians have been successfully applied in shallow learning architectures such as the Support Vector Machine Approach; kernel parameters such as the mean and covariance matrix have a strong statistical meaning for the Gaussian kernel, and last but not least because of their outstanding analytical properties. More detailed studies including comparisons between other kernel functions and distance measures or detailed studies on partially connected RBF networks, for instance, in comparison to CNN-like networks, must be reserved for future work.

## Figures and Tables

**Table 1:** Test accuracy comparison on MNIST.

Model	Test Accuracy	Parameters
DeepRBF	99.50±0.07	229,160
Stochastic Pooling ^b^ [29]	99.53	-
DeepRBF Ensemble ^a^	99.67	1,145,800
CNN + Vector Capsules ^b^ [30]	99.87	1,514,187

^a^ Ensemble of five classifiers. ^b^ CNN architecture.

**Table 2:** Test accuracy comparison on CIFAR10.

Model	Test Accuracy	Parameters
DeepRBF	80.72±0.63	268,600
Stochastic Pooling ^b^ [29]	84.87	-
DeepRBF Ensemble ^a^	85.01	1,343,000
ResNet110 ^b^ [33]	93.57	17,000,000
EfficientNetV2 ^b^ [31]	99.10	121,000,000
ViT-H/14 ^c^ [32]	99.50±0.06	632,000,000

^a^ Ensemble of five classifiers. ^b^ CNN architecture. ^c^ Transformer architecture.

**Table 3:** Test accuracy comparison on EmoDB.

Model	Test Accuracy	Parameters
CNN (MEL spectrograms) [35]	72.06	-
DeepRBF	72.15±1.24	281,040
DeepRBF Ensemble ^a^	74.76	1,405,200
GMM (MFCC) [36]	79.8	-
DeepRBF (binary) ^b^	96.07±0.11	280,400
DeepRBF Ensemble (binary) ^a,b^	96.26	1,402,000

^a^ Ensemble of five classifiers. ^b^ Problem reduced to binary classification task.

**Table 4:** Test accuracy comparison on RAVDESS.

Model	Test Accuracy	Parameters
DeepRBF (speech)	67.36±0.91	281,168
DeepRBF (speech + song)	71.41±1.09	281,168
CNN14 (speech) [37]	72.10 ^b^	79,690,184
DeepRBF Ensemble (speech) ^a^	74.30	1,405,840
ERANN (MEL spectrograms, speech) [38]	74.80 ^b^	24,023,562
DeepRBF Ensemble (speech + song) ^a^	78.82	1,405,840

^a^ Ensemble of five classifiers. ^b^ Four-fold cross-validated results.

## Data Availability

The data used in this study is publicly available. Code is available on request from the corresponding authors.

## Abbreviations

The following abbreviations are used in this manuscript:

RBF	Radial Basis Function
CNN	Convolutional Neural Network
PNN	p-nearest Neighbour
